# Immune and metabolic challenges induce changes in pain sensation and related pathways in the hypothalamus

**DOI:** 10.1152/physiolgenomics.00134.2023

**Published:** 2024-01-08

**Authors:** Sandra L. Rodriguez-Zas, Romana A. Nowak, Adrienne M. Antonson, Laurie Rund, Sreelaya Bhamidi, Andrea N. Gomez, Bruce R. Southey, Rodney W. Johnson

**Affiliations:** ^1^Department of Animal Sciences, University of Illinois at Urbana-Champaign, Urbana, Illinois, United States; ^2^Neuroscience Program, University of Illinois at Urbana-Champaign, Urbana, Illinois, United States; ^3^Division of Nutritional Sciences, University of Illinois at Urbana-Champaign, Urbana, Illinois, United States; ^4^Department of Statistics, University of Illinois at Urbana-Champaign, Urbana, Illinois, United States; ^5^Carl R. Woese Institute, University of Illinois at Urbana-Champaign, Urbana, Illinois, United States; ^6^School of Integrative Biology, University of Illinois at Urbana-Champaign, Urbana, Illinois, United States

**Keywords:** hormones, immune response, RNA sequencing

## Abstract

The hypothalamic molecular processes participate in the regulation of the neuro-immune-endocrine system, including hormone, metabolite, chemokine circulation, and corresponding physiological and behavioral responses. RNA-sequencing profiles were analyzed to understand the effect of juvenile immune and metabolic distress 100 days after virally elicited maternal immune activation during gestation in pigs. Over 1,300 genes exhibited significant additive or interacting effects of gestational immune activation, juvenile distress, and sex. One-third of these genes presented multiple effects, emphasizing the complex interplay of these factors. Key functional categories enriched among affected genes included sensory perception of pain, steroidogenesis, prolactin, neuropeptide, and inflammatory signaling. These categories underscore the intricate relationship between gestational immune activation during gestation, distress, and the response of hypothalamic pathways to insults. These effects were sex-dependent for many genes, such as *Prdm12*, *Oprd1*, *Isg20*, *Prl*, *Oxt*, and *Vip*. The prevalence of differentially expressed genes annotated to proinflammatory and cell cycle processes suggests potential implications for synaptic plasticity and neuronal survival. The gene profiles affected by immune activation, distress, and sex pointed to the action of transcription factors SHOX2, STAT1, and REST. These findings underscore the importance of considering sex and postnatal challenges when studying causes of neurodevelopmental disorders and highlight the complexity of the “two-hit” hypothesis in understanding their etiology. Our study furthers the understanding of the intricate molecular responses in the hypothalamus to gestational immune activation and subsequent distress, shedding light on the sex-specific effects and the potential long-lasting consequences on pain perception, neuroendocrine regulation, and inflammatory processes.

**NEW & NOTEWORTHY** The interaction of infection during gestation and insults later in life influences the molecular mechanisms in the hypothalamus that participate in pain sensation. The response of the hypothalamic transcriptome varies between sexes and can also affect synapses and immune signals. The findings from this study assist in the identification of agonists or antagonists that can guide pretranslational studies to ameliorate the effects of gestational insults interacting with postnatal challenges on physiological or behavioral disorders.

## Graphical abstract

**Figure:**
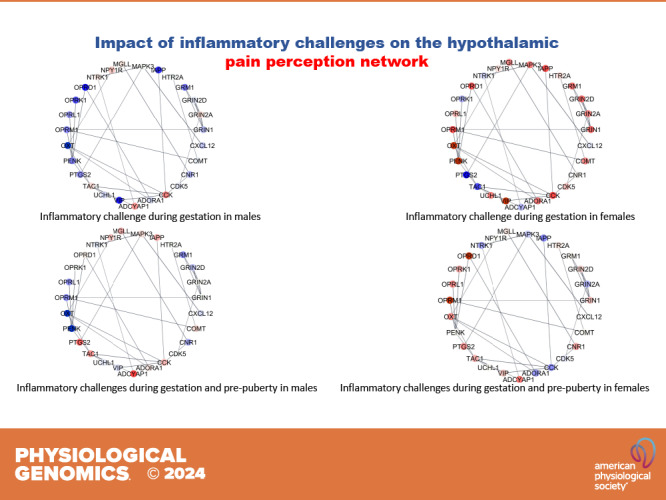

## INTRODUCTION

The two-hit model has advanced the conceptualization of the prolonged effect of immune activation during gestation interacting with environmental insults after birth. Under this model, a first hit or insult to the gestating female, such as an infection, can elicit an immune response and associated signals that disrupt fetal brain structures during development. Gestating rats exposed to lipopolysaccharide challenge presented disruption of inflammatory factors in the TLR4/MYD88/NF-κB pathway (1). This first hit primes the offspring to exhibit a disrupted response to a second hit, such as a postnatal environmental insult. Prenatal insults to brain regions such as the hippocampus and hypothalamus predispose adult offspring toward behavioral and physiological disorders (2).

Studying the hippocampus, a brain structure that plays roles in spatial cognition and memory, we demonstrated that maternal infection with porcine reproductive and respiratory virus (PRRSV) during the last third of gestation was associated with significant changes in the expression of major histocompatibility complex II and inflammatory cytokine genes in the fetal hippocampi 35 days later (3). Adding to the first hit of viral infection, the second hit of weaning at 21 days of age, we detected changes in the hippocampi expression of genes that participate in synthesizing the backbone of terpenoids that have antimicrobial and immunomodulatory properties (4). In consideration of the longer-lasting effects of gestational immune activation (5) and subinflammatory environments (6) in adult rodent hippocampal neurological morphology and function, we studied the interacting effect of prenatal and postnatal inflammatory and metabolic hits on the hippocampus of pigs at 2 mo of age (7). The previous study detected significant changes in the expression of genes annotated to the messenger cAMP and proinflammatory signaling processes.

In rodent and pig models of gestational maternal immune activation (MIA), the offspring display an increased incidence of autism and schizophrenia-like phenotypes, including social deficit, modified activity levels, and sensory-processing sensitivity (8–10). Likewise, postnatal immune challenges, including pro- and anti-inflammatory agents and environmental stressors, can cause behavioral and allodynia alterations (11–13). Although the disruption of molecular mechanisms in the hippocampi has been identified as a contributor to the neurodevelopmental pathologies associated with MIA, less is known of the role of the hypothalamus, another important component of the limbic system, and the two-hit model.

Infection and environmental insults can alter the balance of inflammatory factors impacting the function of the hypothalamus and the systems regulated by this structure, including endocrine and autonomic nervous systems, hormone release and activity cycles, circadian rhythm, and feeding and social behaviors (12). Treatments with agonists of hypothalamus-released products such as oxytocin are effective at modulating inflammation response to viruses such as severe acute respiratory syndrome coronavirus 2 (SARS-CoV-2) (14). Hyperactivity of the hypothalamic-pituitary-adrenal (HPA) axis, typically detected by high peripheral cortisol, has been associated with MIA-related behavioral disorders and allodynia (12, 15). Disruptions of the feed-forward and feed-backward messages and excitatory and inhibitory in the HPA system are associated with disease (16). Therefore, understanding the molecular changes in the inceptive structure in response to insults is a necessary preclinical step.

The link between hypothalamic and behavioral disruption has been reported. NIH Swiss mice exposed to polyinosinic:polycytidylic acid (polyI:C)-induced MIA presented behavioral disruptions and lower expression levels of hypothalamic vasopressin receptor 1a gene compared with control mice (17). Also, among 85-day-old mice exposed to polyI:C-induced MIA, only males displayed increased pain sensitivity and hypothalamic overexpression of the KISS-1 metastasis suppressor (Kiss1) gene (12).

A two-hit model was used to study the effect of postnatal immunological and metabolic insults on the hypothalamic transcriptome of an established model of gestational immune activation elicited by viral inoculation. The changes in expression profile were monitored across ∼17,000 genes in offspring from both sexes exposed to inflammatory signals from the maternal response to PRRSV followed by inflammatory signals from polyI:C or a metabolic state of 24-h fasting 100 days after the first hit. The biological processes and pathways potentially impacted by the two-hit model were identified, and gene network profiling furthered the understanding of the hypothalamic changes.

## MATERIALS AND METHODS

### Experimental Design

The present study was conducted following the guidelines and regulations set forth by the Institutional Animal Care and Use Committee at the University of Illinois using the previously established methodologies (3, 18). Twenty healthy gilts from the PIC Camborough 22 genetic line (PIC, Hendersonville, TN) were inseminated with semen obtained from PIC 359 boars (PIC, Hendersonville, TN). Subsequently, they were transferred from the University of Illinois swine herd to individual chambers within a biosafety facility. The chambers were maintained at a temperature of 22°C, and a 12-h light cycle was followed, with lights on at 7:00 AM. The gilts’ dietary needs were met at each trial stage, and they had unrestricted access to water. At 76 days of gestation, half of the gilts were subjected to gestational immune activation through intranasal inoculation of live PRRSV (strain P129-BV) in a volume of 5 mL, diluted with sterile Dulbecco’s modified Eagle medium (19). The remaining half received an inoculation of sterile medium and served as controls. The PRRSV-challenged group experienced fever and reduced appetite for ∼12 days postinoculation, whereas the control group’s feed intake was restricted to match that of the gestational immune activation group from the previous day (8).

Farrowing was induced by *gestation day 113* and completed in most cases within 24 h, enabling a homogeneous exposure of the pigs to environmental and nutrition conditions (20). Standard inoculations were administered to the offspring to manage iron levels and respiratory diseases, as previously outlined (21). The piglets remained with their mothers in individual crates until they reached 21 days of age. The body weight of the pigs exposed to inflammatory signals during gestation was not significantly different from that of the control group (22). Following that, they were randomly assigned to rooms accommodating four to five piglets each, and a nutritionally complete diet and ad libitum access to water were provided.

Pigs (*n* = 112) of both sex and MIA groups were randomly divided into three juvenile distress groups once the piglets reached 59 days of age. The first group (metabolic distress) underwent a 24-h fasting period, whereas the second group (inflammatory distress) received an intraperitoneal injection of polyI:C (Sigma, St. Louis, MO) at ∼7:00 AM the following day. The polyI:C injection was administered at a dose of 1.0 mg/kg of body weight, following established protocols (23). The remaining third of the pigs were assigned to the saline-treated group, representing the standard condition. These pigs received an intraperitoneal injection of sterile phosphate-buffered saline in a volume equivalent to the polyI:C injection, ensuring a blind treatment assignment.

The 2 × 2 × 3 experimental design is depicted in Supplemental Fig. S1 and included 9 to 10 pigs per group categorized by gestational immune activation (PRRSV-elicited MIA or control), sex (female and male), and juvenile distress (inflammatory distress elicited by polyI:C, metabolic distress of fasting, or saline-treated pigs), respectively. This design enabled the testing of interactions between gestational immune activation and juvenile distress, sex, and juvenile distress, in addition to the effects of each factor.

### Analysis of RNA-Sequencing Data

Established protocols (8) were used to administer intramuscular anesthesia and euthanize the pigs 100 days postexposure to the virally elicited inflammatory signals. The hypothalami of 112 pigs were extracted, flash-frozen, and kept at −80°C until the EZNA kit (Omega Biotek, Norcross, GA) was used to extract RNA. The TruSeq Stranded mRNA preparation kit (Illumina Inc, San Diego, CA) was used to prepare individually and qPCR-quantify RNAseq libraries. The cDNA was sequenced from both ends using the NovaSeq S4 kit and the NovaSeq 6000 sequencer. The bcl2fastq v2.20 software (Illumina Inc, San Diego, CA) was used to demultiplex the resulting reads into FASTQ files that list the sequences and position-based sequences.

Results from evaluating the FASTQ files using the software FASTQC (24) indicated that sequence trimming was unnecessary because of consistent high quality (score Phred > 34) across read positions. The sequences were aligned to the *Sus scrofa* reference genome v11.1, and gene expression abundance was determined using kallisto v0.43.0, following default specifications (25). The trimmed-mean normalized values of genes detected with more than five transcripts per million within each group were subjected to analysis using the edgeR version 3.14 (26).

The gene expression levels were evaluated for the effects of gestational immune activation and sex, each characterized by one contrast (i.e., Control vs. PRSSV and males vs. females, respectively). Also tested were the orthogonal contrasts for juvenile distress (i.e., fasting vs. saline and inflammation vs. saline) and the interactions between the previous factors. The tests that exhibited a false discovery rate-adjusted *P* value (FDR) less than 0.05 (27) were considered statistically significant.

The contrasts of gene expression levels between groups are expressed in log-base2 (fold change). In the model specification, positive values indicate overexpression in Control versus PRRSV, overexpression in males versus females, overexpression in juvenile metabolic distress versus saline, and overexpression in juvenile inflammation distress versus saline. In contrast, negative contrast values denote underexpression for such comparisons. For interactions between stressors, positive values denote antagonistic action between gestational immune activation and either juvenile metabolic or inflammatory distress (i.e., overexpression under either stressor relative to both stressors simultaneously). Conversely, negative interaction values denote synergistic action characterized by overexpression in pigs exposed to gestational immune activation and juvenile distress. For interactions between either stressor and sex, positive values denote overexpression in females from PRRSV gilts, fasting males, or polyI:C-treated males relative to other groups. The raw and normalized expression profiles can be accessed in the National Center for Biotechnology Information GEO dataset repository under the accession GSE245146 (reviewer access token: kjktwiysvhapfgn; https://tinyurl.com/yc4er26p).

### Functional and Network Analyses

Gene Ontology biological processes enriched (FDR < 0.05) with genes impacted by gestational immune activation, juvenile distress, sex, and their interactions were identified using the WebGESTALT package (28). Gene Set Enrichment Analysis (GSEA) enabled the detection of functional categories that had a predominance of genes over- or underexpressed in the respective contrasts. The sign of the GSEA score aligns with the sign of the predominant differential expression pattern. In contrast, the overrepresentation analysis (ORA) enabled to focus processes with a predominance of significant differential expression, irrespective of pattern. The enrichment score in ORA is also a ratio between the observed and expected scores from the gene set, and these values are calculated based on the hypergeometric test (28).

The functional categories tested included the nonredundant Gene Ontology (GO) biological processes (BP) and pathways in the Kyoto Encyclopedia of Genes and Genomes (KEGG). Categories, including five or more differentially expressed genes, are discussed. Preliminary GSEA analysis indicated that the gene annotation to the *Homo sapiens* pathways was more informative than to *Sus scrofa* pathways, and the former genome was used as a reference for all enrichment analyses. The relationship between the enriched pathways was investigated using the Voronoi diagram output from the REACTOME Pathway Analysis Tool (29). The Voronoi diagram approach was selected because of the enhanced visualization of the relationships between pathways through their display as a contiguous area partitioned into broader pathways and more focused subpathways. Also, the pathways are arranged according to the relationships among pathways specified in the event hierarchy (29).

To understand how genes within a biological process are simultaneously affected by gestational immune activation, juvenile distress, and sex, these genes were depicted as nodes in a network. The node color denoted the over- or underexpression pattern in contrast between two groups characterized by the three factors studies: blue indicates underexpression, white indicates even expression, and red indicates overexpression in the first group compared with the second group. The nodes were connected by edges that represent the known relationship between the genes. The molecular relationships were obtained from the STRING v12 database using a confidence score cutoff > 0.7 to enhance true positive and minimize false-positive relations (30). The networks for 12 contrasts between distinct gestational immune activation, juvenile distress, and sex groups were depicted in Cytoscape v. 3.9 (31).

In a similar fashion, the application iRegulon v1.3 was used to study the overrepresentation of transcription factors associated with genes presenting differential expression (FDR < 0.1) in response to gestational immune activation, distress, sex, or interaction effects (32). Genes presenting interaction effects were grouped within the corresponding main effects. The overrepresentation was considered significant when more than 20 target genes presented differential expression for at least one factor, and the normalized enrichment score surpassed a cutoff of 3 that indicates a high probability (cumulative recovery curve area > 0.03) of transcription factor modulation of gene expression (32).

## RESULTS

### Depth and Quality of Sequencing and Differential Expression Metrics

The integrity of the RNA was consistently high (RNA integrity number, RIN, above 7.2) for all 112 hypothalamic samples, and the mean number of sequences per library (sample) was above 165 million reads. The coefficient of variation of the number of reads per sample was ∼0.19, indicating similarity between samples and an even distribution across gestational immune activation, juvenile distress, and sexes. On average, the successful alignment of 77% of the sequences enabled testing the impact of sex, gestational immune activation, and juvenile distress on 16,960 genes in the hypothalamus.

**Figure 1.**
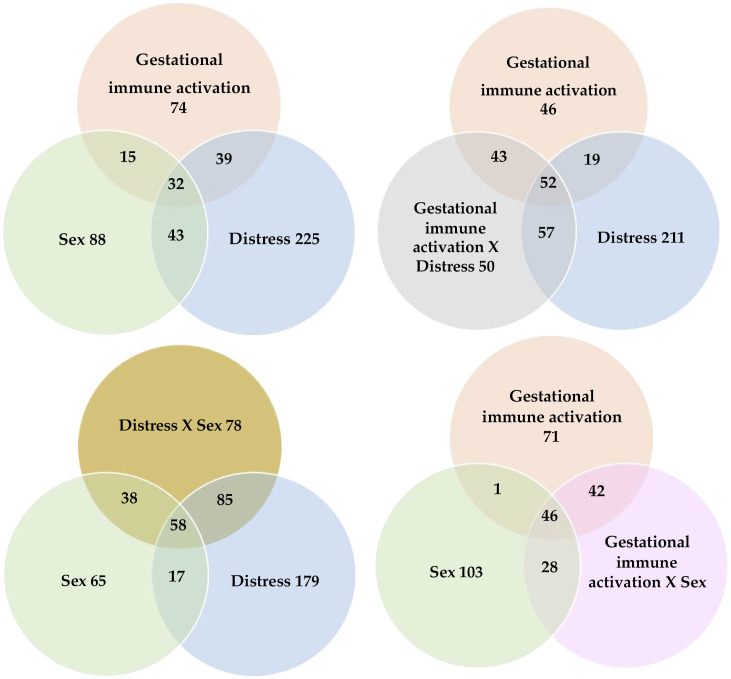
Venn diagrams depicting the number of differentially expressed genes (FDR < 0.05) that share effects of gestational immune activation, juvenile distress, sex, and interactions in the hypothalamus.

Across all six comparisons studied, 1,322 genes exhibited significant differential expression (FDR < 0.05) for at least one main effect or interaction. The effect of the juvenile metabolic and inflammatory distress, alone or interacting with gestational immune activation or sex, accounted for 801 of the differentially expressed genes, followed by the effects of sex (620 genes) and gestational immune activation (544 genes), interacting with the remaining factors or alone. A summary of the genes impacted by the factors studied at FDR < 0.05 and absolute log_2_(fold change) value > 1.5 is available in Supplemental Table S1. Figure 1 depicts the Venn diagrams of the intersection among the counts of genes differentially expressed at FDR < 0.05 among the factors and interactions studied. This figure highlights that separately testing each factor would prevent the detection of the many genes that present significant interactions independent of the main effects. Also, this figure depicts the unique impact that each one of the factors studied can have on the gene profiles in the hypothalamus. The 32 genes that presented gestational immune activation, juvenile distress, and sex effects include ATP synthase peripheral stalk subunit d (*Atp5pd*), corticotropin-releasing hormone (*Crh*), forkhead box G1 (*Foxg1*), glycerophosphodiester phosphodiesterase domain containing 1 (*Gdpd1*), growth hormone 1 (*Gh1*), hemoglobin, peptidase inhibitor 15 (*Pi15*), PR/SET domain 12 (*Prdm12*), PR/SET domain 6 (*Prdm6*), prolactin (*Prl*), parathyroid hormone 2 (*Pth2*), S100 calcium-binding protein A12 (*S100a12*), Sp8 transcription factor (*Sp8*), upstream binding transcription factor-like 1 (*Ubtfl1*), and urocortin 3 (*Ucn3*).

### Gene Pathways in the Hypothalamus Affected by Sex, Gestational Immune Activation, and Juvenile Distress

The overrepresentation and gene set analyses (ORA and GSEA, respectively) of genes presenting interacting and main effects of gestational immune activation, juvenile distress, and sex enable the detection of molecular categories that these factors can impact. The KEGG pathways and Gene Ontology biological processes enriched across two or more factors (FDR < 0.05, |enrichment statistic| > 2) identified by GSEA are listed in Table 1, and Table 2 presents the results from ORA. Complementing the previous lists, an extensive list of KEGG pathways and Gene Ontology biological processes enriched (FDR < 0.05) for one or more factors is included in Supplemental Table S2.

**Table 1.** Normalized enrichment scores of significant (FDR < 0.05; > 5 genes) Gene Ontology biological processes and Kyoto Encyclopedia of Genes and Genomes pathways among genes affected by two or more factors (gestational immune activation, juvenile distress, sex, or interaction) identified using the Gene Set Enrichment Analysis

BP/KEGG	Description	F × G^1^	I × G	S × F	S × I	S × G	G	F	I	S
GO:0001539	Cilium-dependent cell motility	2.4		−1.7	1.9	1.8			−1.9	
GO:0034340	Response to type I interferon	−2.5	−1.8	−2.4					2.1	
GO:0033108	Mitochondrial respiratory chain	−2.0	1.9	1.9					1.7	
GO:0007618	Mating	2.0		2.0		2.1	2.0			
GO:0050886	Endocrine process	1.9		1.7						
GO:0032615	Interleukin-12 production	−1.8	−1.9	−1.9					1.7	
GO:0072350	Tricarboxylic acid metabolic		1.9				1.6			
GO:0007218	Neuropeptide signaling		−2.0	1.8					2.0	
GO:0007631	Feeding behavior			2.3		2.0	1.6			
GO:0019233	Sensory perception of pain			1.9		1.9	1.7			
GO:0001963	Synaptic transmit, dopaminergic			1.8		2.0	1.7			
GO:0007215	Glutamate receptor signaling			2.1			1.8			
GO:0070972	Protein to endoplasmic reticulum	−2.1								2.3
GO:0019098	Reproductive behavior	2.0		1.7		2.2	2.0			
GO:0035902	Response to immobilization stress	1.8				1.8	1.7			
hsa03010	Ribosome	−2.5			−1.9		−1.8	2.4	2.4	2.7
hsa05144	Malaria	−2.2	−2.2	−2.1			−2.2	2.0	2.6	
hsa00190	Oxidative phosphorylation	−2.1			−2.2			2.2	2.5	
hsa04657	IL-17 signaling	−1.9	−1.9		1.7				2.9	
hsa04622	RIG-I-like receptor signaling	−1.9			1.7				2.1	
hsa04932	Nonalcoholic fatty liver disease	−1.7							2.1	
hsa01230	Biosynthesis of amino acids		2.2			2.1	2.2			
hsa04668	TNF signaling		−2.1						3.0	
hsa05323	Rheumatoid arthritis		−2.1						2.4	
hsa04064	NF-κB signaling		−1.9	−1.7	1.9				2.6	
hsa04917	Prolactin signaling					1.9			2.1	
hsa04913	Ovarian steroidogenesis			−1.7	−2.1				2.2	

^1^Enrichment sign interpretation of contrast. F, juvenile fasting - saline; F × S, fasting male - fasting or male; G, gestational activation - control; G × F, gestational and fasting - either one stressor; G × I, gestational and polyI:C- either one stressor; G × S, gestational immune activation and male - either one condition; I, juvenile polyI-C - saline; I × S, polyI:C male - polyI:C or male; S, males - females. A negative (positive) sign denotes that genes in the category are underexpressed (overexpressed) on the left side relative to the right side group of the contrast (−).

**Table 2.** The enrichment ratio of Kyoto Encyclopedia of Genes and Genomes pathways and Gene Ontology biological processes in the hypothalamus enriched (FDR < 0.05; > 5 genes in a category, enrichment score > 5) identified using the overrepresentation analysis among genes presenting significant (FDR < 0.1) for the effects of gestational immune activation, juvenile distress, sex, or interaction effects

BP/KEGG	Description	D × G^1^	S × D	S × G	G	D	S
GO:0007631	Feeding behavior	11.4	9.8	17.7	14.5	8.9	16.3
GO:0007218	Neuropeptide signaling	7.7	8.8			7.4	12.8
GO:0032570	Response to progesterone					10.9	
GO:0021536	Diencephalon development	10.2	10.2			5.4	
GO:0035270	Endocrine system development	6.1	9.5				11.6
GO:0035902	Response immobilization stress	16.5		20.6	12.2		
GO:0051412	Response to corticosterone	25.3			26.3	33.7	
GO:0061844	Antimicrobial immune response	11.6			11.1		
GO:0071357	Response type I interferon	9.2				7.9	
GO:0045071	Negative regulation viral replication	12.9				9.9	
GO:0042446	Hormone biosynthetic process				6.8		
GO:0042698	Ovulation cycle				7.2		
GO:0045834	Positive regulation lipid metabolism					4.9	
GO:0032368	Regulation of lipid transport					5.7	
GO:2000351	Regulation endothelial cell apoptosis					8.1	
hsa04657	Interleukin-17			11.0			
hsa04917	Prolactin			10.5			
hsa04064	Nuclear factor-κB			8.5			
hsa05134	Legionellosis				6.7		

^1^Enrichment ratio. D, juvenile distress; G, gestational immune activation; D × G, interaction between juvenile distress and gestational immune activation; S, sex; S × D, interaction between sex and juvenile distress; S × G, interaction between sex and gestational immune activation.

The GSEA results in Table 1 denote the pattern of the genes in the molecular category according to profiles from more to less differentially expressed and the sign of the normalized enrichment score (NES). Positive NES indicates genes overexpressed in Control relative to PRRSV, under either juvenile distress relative to saline, and males relative to females, whereas negative signs correspond to the reverse profile. For interactions, positive NES indicates gene overexpression in pigs exposed to one of the two stressors (gestational immune activation or juvenile distress), females from PRRVS gilts, or juvenile-distressed males. Conversely, for interaction, negative NES indicates gene overexpression in pigs exposed to both stressors (gestational immune activation or juvenile distress), males from PRRVS-treated gilts, or juvenile-distressed females.

The preponderance of negative NES for the interaction between gestational immune activation and both juvenile distresses among immune-related categories (e.g., hsa04657 interleukin-17 signaling pathway; GO:0032615 interleukin-12 production). This result is further confirmed in Supplemental Table S2 (e.g., hsa04514 cell adhesion molecules or CAMs; hsa05133 Pertussis). The negative NES across juvenile distresses indicates that the genes were overexpressed in the hypothalamus of pigs exposed to gestational and juvenile treatments.

Notably, the NES signs were opposite for the interaction between sex and both juvenile distresses, and more categories reached FDR < 0.5 for the interaction between sex and fasting than sex and inflammation (Table 1). The previous findings suggest that immune-related genes were overexpressed in fasting females and polyI:C-treated males compared with other combinations. In contrast, genes annotated to neuronal signaling processes (e.g., GO:0019233 sensory perception of pain and GO:005088 endocrine process) were overexpressed in juvenile fasting males compared with other combinations.

A positive NES sign was detected for behavior and sensory processes (e.g., GO:0019233 sensory perception of pain; GO:0007618 mating; GO:0007631 feeding behavior) enriched for the interaction between gestational immune activation and sex and for gestational immune activation as the main effect. The NES signs indicate that genes in these categories are overexpressed in females exposed to gestational immune activation compared with other combinations (Table 1).

Interestingly, although many molecular categories were enriched for fasting distress interacting with gestational immune activation and sex, few categories reached significant enrichment for fasting distress alone. Most categories enriched in response to fasting distress alone were associated with immune response (e.g., hsa00190 oxidative phosphorylation and hsa05144 Malaria). All KEGG pathways and biological processes except one (GO:0001539 cilium or flagellum-dependent cell motility) were enriched for the effect of inflammation distress and presented a positive NES sign, including immune and signaling (synaptic and hormonal) categories. This finding indicates that the inflammatory agent upregulated the genes in these distinct categories (Table 1). Although many enriched functional categories were detected for the effect of sex (Supplemental Table S2), the two categories in Table 1 reflect that sex alone impacted processes distinct from the effect interacting with gestational or juvenile distress conditions.

The study of the molecular categories enriched among the top differentially expressed genes (FDR < 0.5) using ORA added integral information on the effects of gestational immune activation, juvenile distress, and sex on the hypothalamus pathways. Consistent enrichment in immune response, neuronal signaling, and behavioral processes was detected by the ORA and GSEA approaches (e.g., GO:0007218 neuropeptide signaling; GO:0007631 feeding behavior; hsa04917 prolactin; hsa04657 interleukin-17 signaling; hsa04668 tumor necrosis factor signaling). Similar to the GSEA results, the effect of juvenile distress, alone or interacting with sex and gestational immune activation, had the highest number of enriched categories (Table 2).

Notable results from ORA were the multiple categories associated with the developmental processes, such as GO:002153 diencephalon development and GO:002151 spinal cord development (Table 2, Supplemental Table S3). Another finding from the ORA analysis was the enrichment of categories associated with the response to stress, such as GO:0051412 response to corticosterone response, GO:0035902 response to immobilization stress, and GO:0009409 response to cold (Table 2, Supplemental Table S3). The stress-related pathways and processes were enriched among genes differentially expressed in response to metabolic and inflammatory distress.

**Figure 2.**
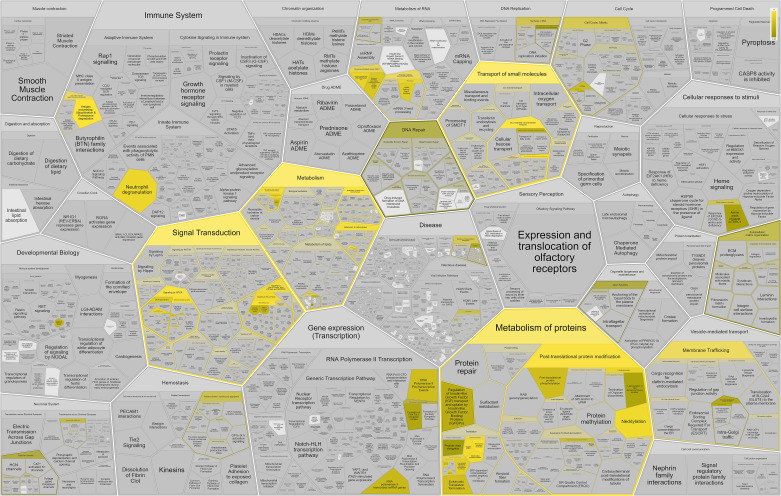
Visualization of the enrichment analysis using the Reactome relational knowledgebase of biomolecular pathways.

The relative enrichment and relationship between the pathways overrepresented among genes presenting differential gene expression (FDR < 0.05) were visualized using the REACTOME knowledgebase (Fig. 2). The enriched REACTOME categories were aligned with the results from the GSEA and ORA analyses, including the immune system, signal transduction, metabolism, cell cycle, metabolism of proteins, and transport of small molecules. The signal transduction pathway includes the subpathway of G-protein-coupled receptor processes that interact with dopamine and corticosteroids. The metabolism pathway encompasses the metabolism of lipids, and the immune system pathway includes the interferon and prolactin-signaling processes. The metabolism of protein pathway includes the peptide hormone metabolism, immune system, prolactin, neuropeptide signaling, and the neuronal system, such as the glutamate-loaded synaptic vesicle process.

### Gene Profiles in the Hypothalamus Affected by Sex, Gestational Immune Activation, and Juvenile Distress

The profiles of genes that participate in the enriched functional categories were studied to understand the interplay between gestational immune activation, juvenile distress, and sex. The fold change (in logarithm base two units) of genes in sensory perception of pain, immune response, and prolactin-signaling pathways presenting significant differential expression (FDR-adjusted *P* value < 0.05, |fold change| > 1.5) were analyzed (Table 3). Complementing the previous profiles, Supplemental Table S1 lists all genes impacted at FDR < 0.05 and minimum |fold change| > 2.8. Table 3 lists differentially expressed genes, including gene symbol, name, profile, and statistical significance.

**Table 3.** Differential expression (log2(fold change)) of genes in the hypothalamus impacted (FDR-adjusted P value < 0.05, |log2(fold change)| > 1.5) by gestational immune activation, juvenile distresses, sex, and interactions that participate in the functional categories of sensory perception, immune response, or prolactin-signaling pathways

Gene Category and symbol	G × F^1^	G × I	S × F	S × I	G × S	G	F	I	S
SENSORY ^2^									
*Vip*	2.5	0.8	3.6	2.9	2.0		−1.5	−0.5	−2.7
*Ptgs2*							0.1	3.0	
*Prdm12*	2.7	1.5	2.9	1.7	3.9	1.9	−1.6	−1.3	−2.2
*Oxt*	3.4	1.2	1.5	1.6	4.6	1.7			−2.2
*Oprd1*			1.6	0.4	1.6				
*Ntsr1*			−0.8	−1.5					
IMMUNE									
*Ccl2*							0.4	3.3	
*Cxcl8*	−3.4	−2.5	−3.4	1.3			3.4	7.7	
*Ifi6*	−2.1	−0.4							
*Bst2*	−2.2	−0.7							
*Isg15*	−2.3	−0.5					0.4	1.6	
*Isg20*	−4.2	−0.9	−0.6	2.9					−1.4
*Oasl*	−3.4	−1.9				−2.0	−0.3	1.2	
*Rsad2*	−2.4	−2.1	−2.1	0.6			0.6	2.2	
*Ccl4*							0.5	4.3	
*Cxcl10*			−0.9	0.8			0.2	3.3	
*Il18*						−1.3			
*Nfkbia*							0.0	1.5	
*Zbp1*			−1.6	0.3					
PROLACTIN									
*Cga*	1.1	−1.2	−3.9	−5.3			1.4	4.7	3.6
*Cish*							−0.1	1.1	
*Fos*							−0.7	2.6	
*Irf1*							−0.1	1.9	
*Lhb*	1.6	−0.3			2.0		−0.3	1.1	
*Prl*	2.7	2.0	−5.6	−4.7	4.0	2.5	0.9	3.9	4.0
*Socs1*							−0.1	2.0	
*Socs3*							0.1	3.3	
*Prlr*			1.7	3.0	2.9		−1.0	−1.8	−2.3
*Th*			−1.9	−1.0		2.0			

^1^G = log2(control - gestational immune activation). F, log2(fasting - saline); F × S, log2(fasting male –fasting or male); G × F, log2(fasting or gestational activation - both stressors); G × I, log2 (polyI:C or gestational activation - both stressors); G × S, log2(gestational immune activation and female - gestational activation or female); I, log2(polyI:C - saline); I × S, log2(polyI:C male - polyI:C or male); S = log2(males - females). Negative (positive) log2(fold change) denotes gene underexpression (overexpression) on the left side relative to the right side group within the contrast (−).

^2^Sensory perception category genes: vasoactive intestinal peptide (*Vip*), prostaglandin-endoperoxide synthase 2 (*Ptgs2*), PR/SET domain 12 (*Prdm12*), oxytocin/neurophysin I prepropeptide (*Oxt*), opioid receptor delta 1 (*Oprd1*), neurotensin receptor 1 (*Ntsr1*); immune response category genes: chemokine (C-C motif) ligand 2 (*Ccl2*), C-X-C motif chemokine ligand 8 (*Cxcl8*), interferon alpha inducible protein 6 (*Ifi6*), bone marrow stromal cell antigen 2 (*Bst2*), interferon-stimulated exonuclease gene 15 (*Isg15*), interferon-stimulated exonuclease gene 20 (*Isg20*), 2′-5′-oligoadenylate synthetase like (*Oasl*), radical *S*-adenosyl methionine domain containing 2 (*Rsad2*), chemokine (C-C motif) ligand 4 (*Ccl4*), C-X-C motif chemokine ligand 10 (*Cxcl10*), interleukin 18 (*Il18*), nuclear factor kappa B inhibitor alpha (*Nfkbia*), Z-DNA binding protein 1 (*Zbp1*); prolactin signaling pathway genes: glycoprotein hormones, alpha polypeptide (*Cga*), cytokine-inducible SH2 containing protein (*Cish*), fos proto-oncogene, AP-1 transcription factor subunit (*Fos*), interleukin 1 beta (*Il1b*), interferon regulatory factor 1 (*Irf1*), luteinizing hormone subunit beta (*Lhb*), prolactin (*Prl*), suppressor of cytokine signaling 1 (*Socs1*), suppressor of cytokine signaling 3 (*Socs3*), prolactin receptor (*Prlr*), tyrosine hydroxylase (*Th*).

Several consistent profiles can be identified among genes within functional categories and factor tested (Table 3). In the sensory perception of pain category, the genes (e.g., *Vip*, *Prdm12*, and *Oxt*) were overexpressed in pigs exposed to gestational immune activation, and both types of juvenile distress relative to controls. The previous genes were also overexpressed in females relative to males, females exposed to gestational immune activation, or males exposed to juvenile distress or relative to either condition alone.

In the immune response category, the genes (e.g., *Cxcl8*, *Isg20*, *Rsad2*, *Cxcl10*, and *Zbp1*) were underexpressed in pigs exposed to gestational immune activation and both types of juvenile distress relative to controls. The previous genes were also underexpressed in females relative to males, males exposed to metabolic distress, and females exposed to inflammation distress or relative to either condition alone. In the prolactin-signaling category, the genes (e.g., *Fos*, *Irf1*, *Lhb*, *Prl*, *Socs1*, and *Socs3*) were overexpressed in males relative to females and in pigs exposed to gestational immune activation and inflammation distress relative to the conditions.

### Network Characterization of the Impact of Sex, Gestational Immune Activation, and Juvenile Distress on Genes in the Hypothalamic Sensory Perception of Pain Pathway

The analysis of enriched biological categories uncovered a cluster of processes, including genes that presented similar patterns in response to gestational immune activation, juvenile stressors, and sex (Table 1). These processes included sensory perception of pain (GO:0019233), dopaminergic synaptic transmission (GO:0001963), glutamate receptor signaling (GO:0007215), exploration behavior (GO:0035640), and feeding behavior (GO:0007631). Although the expression patterns include interaction among the factors studied, the overarching profile includes underexpression in pigs that experienced gestational immune activation relative to control, particularly among males from gestational immune activation gilts, and overexpression in fasting males, relative to other groups sharing characteristics. The same patterns were detected in genes annotated to anesthetic (GO:0072347), reproductive behavior (GO:0019098), and mating (GO:0007618) in addition to overexpression in fasting pigs from control gilts.

**Figure 3.**
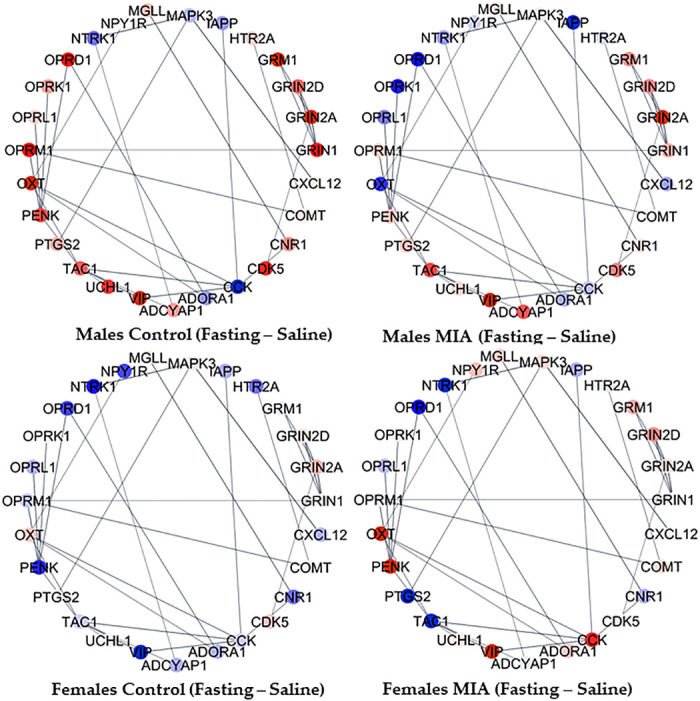
Sensory perception of pain (GO:0019233) network of hypothalamic gene profiles between juvenile fasting distress and saline within gestational immune activation and sex groups. Genes are portrayed by nodes, and STRING repository relationships are portrayed by lines. Extremes of the node color scheme are red, representing overexpression in the first group in relation to the second group, white representing evenly expressed between groups, and blue representing underexpression in the first group in relation to the second group.

**Figure 4.**
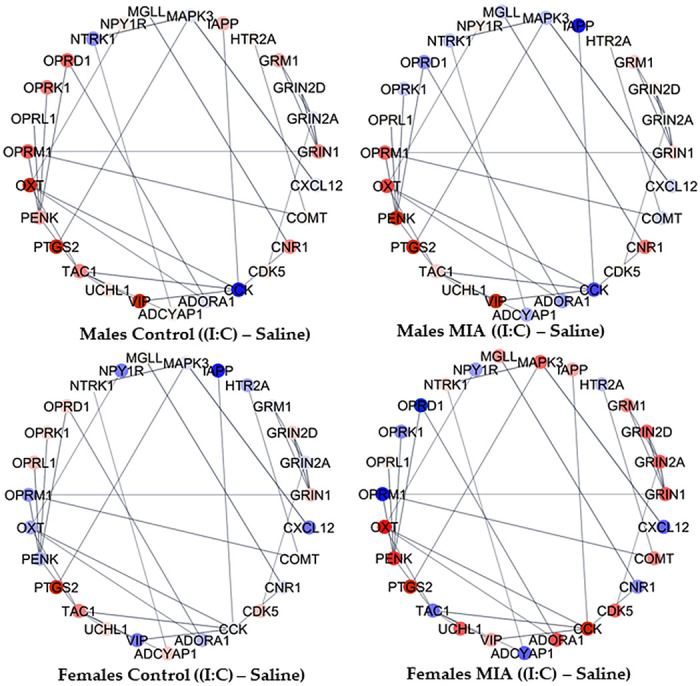
Sensory perception of pain (GO:0019233) network of hypothalamic gene profiles between juvenile polyI:C inflammatory distress and saline within gestational immune activation and sex group. Genes are portrayed by nodes, and STRING repository relationships are portrayed by lines. Extremes of the node color scheme are red, representing overexpression in the first group in relation to the second group, white representing evenly expressed between groups, and blue representing underexpression in the first group in relation to the second group.

**Figure 5.**
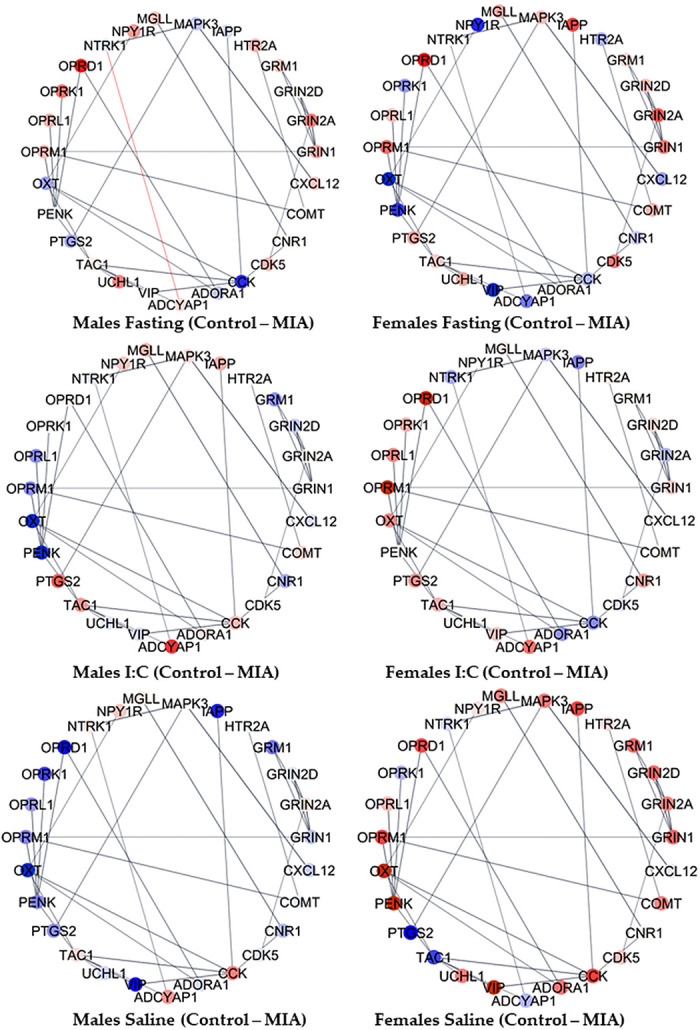
Sensory perception of pain (GO:0019233) network of hypothalamic gene profiles between gestational immune activation (MIA) and control groups with sex and juvenile distress groups. Genes are portrayed by nodes, and STRING repository relationships are portrayed by lines. Extremes of the node color scheme are red representing overexpression in the first group in relation to the second group, white representing evenly expressed between groups, and blue representing underexpression in the first group in relation to the second group.

The complexity of the interplay between gestational immune activation, juvenile distress, and sex simultaneously on multiple genes in the cluster of enriched processes was evident in the network of genes in the sensory perception of pain category. To facilitate the visualization of notable relationships between genes, 26 genes with the highest variability in fold change between groups among the 42 genes annotated to the sensory perception of pain were depicted in networks. The 26 informative genes included were adenylate cyclase-activating polypeptide 1 (*Adcyap1*), adenosine A1 receptor (*Adora1*), adrenoceptor alpha 2 C (*Adra2c*), cholecystokinin (*Cck*), cholinergic receptor nicotinic alpha 4 subunit (*Chrna4*), cannabinoid receptor 1 (*Cnr1*), C-X-C motif chemokine ligand 12 (*Cxcl12*), glutamate ionotropic receptor NMDA type subunit 2 A (*Grin2a*), glutamate metabotropic receptor 1 (*Grm1*), homeobox D1 (*Hoxd1*), islet amyloid polypeptide (*Iapp*), mitogen-activated protein kinase 3 (*Mapk3*), neuropeptide FF-amide peptide precursor (*Npff*), neuropeptide Y receptor Y1 (*Npy1r*), neurotrophic receptor tyrosine kinase 1 (*Ntrk1*), neurotensin receptor 1 (*Ntsr1*), opioid receptor delta 1 (*Oprd1*), opioid receptor kappa 1 (*Oprk1*), opioid-related nociceptin receptor 1 (*Oprl1*), opioid receptor mu 1 (*Oprm1*), oxytocin/neurophysin I prepropeptide (*Oxt*), proenkephalin (*Penk*), PR/SET domain 12 (*Prdm12*), prostaglandin-endoperoxide synthase 2 (*Ptgs2*), tachykinin precursor 1 (*Tac1*), and vasoactive intestinal peptide (*Vip*). Figures 3 and 4 portray the gene network profiles when comparing the metabolic and inflammatory stressors relative to saline within gestational immune activation and sex groups. Figure 5 portrays the gene network profiles when comparing the gestational immune activation relative to the control group within the juvenile stressor and sex groups. Within the Cytoscape framework and STRING meta-repository of molecular interactions, interactions supported at ≥70% index across the text mining, coexpression, and experimental databases are depicted. The continuous color scheme ranges from blue (underexpression in the first relative to the second group contrasted) to white, denoting even expression, and red (overexpression in the first relative to the second group contrasted).

The networks of genes associated with pain perception, dopaminergic synapses, and glutamate signaling highlighted how exposure to gestational immune activation modulates the impact of juvenile stress in the hypothalamus and varies between sexes. Figure 3 highlights that *Oprd1* was underexpressed in fasting compared with saline-treated pigs across all groups except for males from control mothers, and *Vip* was overexpressed in fasting relative to saline-treated pigs except for females from control mothers. Genes in the glutamate ionotropic receptor NMDA (GRIN) family were consistently overexpressed in fasting males from control mothers. Among the genes presenting more stable patterns, *Tac1* is overexpressed in fasting males, whereas *Ntrk1* is underexpressed in fasting relative to saline-treated pigs. Also, *Iapp* was consistently overexpressed in fasting compared with saline pigs, with an extreme differential expression among males from PRRSV-inoculated mothers.

Figure 4 showcases the interplay between gestational and juvenile inflammatory stresses within sex. For example, *Penk* was overexpressed, whereas *Oprd1* was underexpressed in pigs exposed both to gestational activation and juvenile polyI:C compared with pigs exposed to either one or no challenge. Likewise, *Vip*, *Cnk1*, and opioid receptor mu 1 (*Oprm1*) were overexpressed, whereas *Cck* was underexpressed in polyI:C-challenged males compared with all other pig groups. Genes in the GRIN family (e.g., *Grin1*, *Grin2A*) were consistently overexpressed in polyI:C females exposed to gestational immune activation, *Oxt* was overexpressed in the polyI:C relative to saline-treated pigs (except for control females), and *Ptgs2* was overexpressed in the polyI:C-challenged pigs compared with fasting or saline-treated pigs.

The lingering effect of gestational immune activation related to juvenile stress and sex on the gene networks is demonstrated in Fig. 5. Notably, *Cck* was overexpressed in the hypothalamus of pigs from gestational immune activation gilts relative to control pigs in animals exposed to juvenile stress, irrespective of the type of stress. Also, *Oprm1* and *Oprd1* were underexpressed in pigs from PRRSV-challenged relative to control mothers except for saline-treated males. Gestational immune activation was associated with the overexpression of *Ptgs2* in polyI:C-challenged pigs compared with the saline-treated counterparts. The overrepresentation analysis of the transcription factors that correspond to genes differentially expressed (FDR < 0.1) between gestational immune activation, distress, and sex groups is presented in Table 4. A notable finding is that most of the transcription factors are enriched for one of the factors studied, with the exception of SHOX2 (SHOX homeobox 2), SUZ12 polycomb repressive complex 2 subunit (SUZ12), C-terminal binding protein 2 (CTBP2), and RE1 silencing transcription factor (REST).

**Table 4.** Transcription factors associated with 20 or more target genes presenting differential expression in response to gestational activation (G), juvenile distress (D), sex (s), or interactions

Transcription Factor	G	D	S
SHOX2	105	71	
STAT1	82		
PAX7	45		
DDX4	41		
NFKB1	44		
SUZ12	57	48	50
CTBP2	20	18	41
REST	18	43	54
BSX			52
DLX5			55
OTP			47
JAZF1			55
GATA3		75	
RAD21		35	

## DISCUSSION

The hypothalamic transcriptome responses to maternal immune activation in utero, followed by inflammatory and metabolic distress at a juvenile age, and the variations between sexes were investigated. Using a model of maternal infection with porcine reproductive and respiratory virus during the final third of gestation enabled an understanding of the impact of inflammatory signals while the offspring’s hypothalamus is still differentiating. Advancing the understanding of the “two-hit” hypothesis, female and male offspring exposed to the gestational immune activation were subsequently exposed to either inflammatory distress elicited by the viral mimetic polyI:C or metabolic distress elicited by fasting at 60 days of age or ∼100 days after the prenatal viral challenge.

More than 1,300 genes presented significant (FDR < 0.05) gestational immune activation, juvenile distress, and sex effects (Supplemental Table S1), and the intersection of these effects is summarized in the Venn diagram series in Fig. 1. Among the differentially expressed genes, 33% presented more than one effect. Likewise, genes presenting interacting effects varied between 42% and 51% of all genes presenting either of the main effects. These results confirm the need to study the prolonged effects of gestational immune activation and the effects of inflammatory or metabolic distress in the context of gestational immune activation. Our findings also confirm that the sex of the individual plays an important role in the impact of the challenges in the hypothalamus.

Many genes impacted by the effects of gestational immune activation and distress are targets of transcription factor SHOX2. The potential modulation of SHOX2 by the prenatal and postnatal inflammatory conditions studied is consistent with reports of the participation of this molecule in neural maintenance or transmission and in processing somatosensory information (33). Also, the expression of SHOX2 in the rat hippocampus increases one day after administration of the inflammatory agent LPS (34). Most of the overrepresented transcription factors were uniquely associated with differentially expressed genes for one of the three effects studied, such as GATA3, brain-specific homeobox (BSX), and STAT1. In the present study, the Gata3 gene was overexpressed in the hypothalamus of 2-mo-old pigs exposed to MIA or postnatal polyI:C. Also, GATA3 and STAT1 were enriched in the hippocampus and hypothalamus of younger pigs exposed to MIA and postnatal stressors (35).

Three major functional categories were identified among the KEGG pathways and GO biological processes encompassing a significant (enrichment FDR < 0.05) number of genes affected by sex, gestational immune activation, and juvenile distress. The functional groups include *1*) sensory perception and neuroendocrine signaling, *2*) inflammatory response, and *3*) cellular cycle-related mechanisms (Tables 1 and 2). The enrichment of these groups of categories is supported by reports that inflammatory mechanisms disrupted by gestational immune activation, such as those elicited in rats, mice, and pigs, are associated with diverse reproductive and behavioral disorders (23, 36).

The enriched categories among the differentially expressed genes encompassed sensory perception, neuroendocrine, and inflammatory signaling including the GO biological processes and KEGG pathways of sensory perception of pain, dopaminergic synaptic transmission, glutamate receptor signaling, neuropeptide signaling, cilium-dependent cell motility, response to corticosterone and immobilization stress, feeding behavior, ovulation cycle, steroidogenesis, endocrine system development and process, response to progesterone hormone, prolactin signaling, and reproductive behavior.

### Sensory Perception of Pain Process, Neuropeptide Genes, and Networks in the Hypothalamus Affected by Sex, Gestational Immune Activation, and Juvenile Distress

Enriched mechanisms that participate in sensory perception of pain, dopaminergic synaptic transmission, glutamate receptor signaling, and associated behaviors encompass genes underexpressed in pigs exposed to gestational immune activation and sex-dependent patterns (Table 1). Immune activation during gestation and behavioral disorders in the offspring later in life has been associated with disrupted mechanical allodynia and tactile sensory perception (37). The gestational immune activation and sex-dependent pattern observations are also consistent with reports that gestational immune activation elicited by polyI:C resulted in altered pain sensitivity around puberty and accelerated time-to-puberty in mice (12). Mice exposed to polyI:C during gestation presented higher pain sensitivity after birth. Moreover, earlier age at first estrus was correlated with a higher mechanical allodynia threshold at age P45, while the correlation was absent in females and became negative in males by age P70 (12). Some behaviors can be monitored as proxy measurements of pain perception, and pigs exposed to MIA had lower rates of social behaviors, such as playing at 60 days of age (23). Also, the increased lethargy after polyI:C treatment was more marked in MIA females than in MIA males and pigs in the Control group.

In the sensory perception of pain pathway, gene *Prdm12* codes for a transcription factor involved in sensory neuron development and proper nociceptive responses in humans and rodents (38). Also, individuals with mutations present insensitivity to pain. Consistent with the profile observed in the present study (Table 3), *Prdm12* was underexpressed in the anterior hypothalamus of 28-day-old male offspring of female mice exposed to Bisphenol A (BPA) during gestation (39).

The network of genes in the sensory perception of pain process depicts the response of opioid receptors *Oprm1* and *Oprd1*, both underexpressed in the hypothalamus of pigs exposed to gestational immune activation, irrespective of juvenile distress or sex (Figs. 3, 4, and 5). The profile depicted in the networks is consistent with reports that neonatal lipopolysaccharide (LPS) challenge in rats was associated with underexpression of *Oprm1* in the prefrontal cortex in both sexes and increased mechanical pain sensitivity (40). Also, *Oprd1* has been proposed as a promising target for managing pain (41).

The overrepresentation of the neuropeptide signaling process detected in the present study is aligned with the reported role of neuropeptide signaling in the interconnection between sensory perception of pain, endocrine, and metabolic processes. Disruption in the expression of transcripts of neuropeptide prohormone and receptor genes was associated with sensitivity to pain in mice’s cephalic region and hind paws (42). Also, neuropeptide levels are regulated by metabolic hormones and influence the secretion of gonadotropin-releasing hormone (GnRH), luteinizing hormone (LH), feeding, and metabolism (43). The enrichment of neuropeptide signaling was characterized by underexpression in pigs exposed to gestational immune activation and overexpression in metabolic-stressed males (Table 1). The previous neuropeptide gene profiles were also detected in the pituitary gland of 3-wk-old pigs, including overexpression in males exposed to weaning stress that can be accompanied by temporary lower feed consumption and underexpression in pigs exposed to gestational immune activation (44). Similarly, changes in neuropeptide gene expression observed in the hypothalamus of rats exposed to metabolic stress have been associated with neural regulatory, behavioral, and physiological adaptations (45).

Among the neuropeptide genes, gestational immune activation also impacted the expression of *Adcyap1*, which produces neuropeptides PACAP27 and PACAP38. In agreement with this result, gestational immune activation impacted the levels of *Adcyap1* transcripts in the hippocampus and amygdala of 3-wk-old pigs (46). *Adcyap1* knockout lines have diminished mechanical hyperalgesia and lower chronic pain response (47). Also, neuropeptide gene *Cck* was overexpressed in the hypothalamus of pigs exposed to gestational immune activation, irrespective of the type of stress. Similarly, *Cck* was upregulated in a meta-analysis of brain gene expression data from mouse models of gestational immune activation using polyI:C (48).

The hypothalamic profile of neuropeptide gene *Vip* in response to gestational immune activation was opposite between the sexes, underexpressed in males (overexpressed in females), and not exposed to a second hit (Fig. 5). Also, *Vip* was overexpressed in the hypothalamus of males exposed to juvenile distresses relative to saline-treated pigs (Figs. 3 and 4). Consistent with the network profile, *Vip* transcript isoforms were underexpressed in the nucleus accumbens of male mice, presenting mechanical sensitivity to pain elicited by nitroglycerin treatment in the cephalic and hind paw regions (42). Similarly, the prefrontal cortex of male mice exposed to LPS-induced gestational immune activation reported lower density and sparse distribution of VIP+ neurons, and this effect was enhanced under postnatal hypoxia stress (49). The response of *Vip* to gestational immune activation is aligned with reports that *Vip* is underexpressed in a postmortem transcriptome study of the dorsolateral prefrontal cortex of adults diagnosed with schizophrenia spectrum disorder, which is associated with gestational immune activation (50).

Neuropeptide gene *Penk* was overexpressed in polyI:C-treated juvenile pigs exposed to gestational immune activation. This pattern is consistent with the overexpression of a *Penk* transcript in the nucleus accumbens of mice presenting hypersensitivity to cephalic and hind paw pain due to nitroglycerin treatment (42). Neuropeptide gene *Oxt* was overexpressed in females and males exposed to maternal immune activation and subsequently challenged with polyI:C relative to the saline (Fig. 4). In agreement with the previous network pattern, *Oxt* was overexpressed in the pituitary gland of 3-wk-old pigs exposed to gestational immune activation and weaning stress (44). Likewise, OXT agonists are effective modulators of virally induced inflammatory signals (14).

Several genes in the sensitivity to pain network were differentially expressed in association with fasting. Neuropeptide gene *Tac1* is overexpressed in fasting relative to saline males irrespective of gestational immune activation, *Ntrk1* is underexpressed in fasting relative to saline across all gestation and sex groups, and neuropeptide gene *Iapp* was consistently overexpressed in the fasting compared with the saline group, with an extreme differential expression among gestationally activated males. Reduction in feed intake can be observed in response to environmental cues that activate stress-related pathways, such as weaning with reported changes in peripheral markers (51). Consistent with our findings, *Tac1* and *Iapp* regulate food intake (52, 53). The gene patterns observed in the present study may be aligned with reports that anorexia is associated with reduced pain sensitivity and fasting was associated with higher brain levels of serotonin, endogenous opioids, and endocannabinoids, and induced neuroendocrine activation and modest cellular stress response with increased release of neurotrophic factors (54).

### Synaptic Transmission Processes in the Hypothalamus That Are Affected by Sex, Gestational Immune Activation, and Juvenile Distress

The underexpression of genes in the hypothalamus exposed to gestational immune activation within the enriched sensory perception of pain was positively correlated with the underexpression of genes in the enriched dopaminergic synaptic transmission and glutamate receptor signaling (Table 1). Similarly, the expression of glutamate receptor genes was lower in the brains of patients with schizophrenia spectrum disorder, another neurodevelopmental disorder associated with gestational immune activation (55). Aligned with our findings, disruption in the expression of genes that participate in mechanical allodynia follows that of genes annotated to the endogenous opioid system and stress responses (56). Transcriptome studies of the nucleus accumbens of mice presenting mechanical hyperalgesia elicited by opioids also identified the underexpression of genes within enriched neuronal synaptic processes (57).

The interaction between gestational immune activation and sex among the genes in the dopaminergic synaptic transmission and glutamate receptor signaling processes is characterized by more extreme underexpression in males exposed to gestational immune activation. Consistent with the observed patterns, adult male mice exposed to polyI:C-induced gestational immune activation had significantly decreased levels of dopamine receptors in the prefrontal cortex (58). The previous profile associated with gestational immune activation has been proposed as a compensatory mechanism for enhanced dopamine levels (59), and the changes in dopamine pathways have been connected with the increased risk of drug addiction by patients with autism spectrum disorder (60).

Among the genes in the enriched glutamate receptor signaling process is *Th*, which codes for the rate-limiting enzyme of dopamine synthesis. *Th* was underexpressed in the hypothalamus of pigs exposed to gestational immune activation and in juvenile males exposed to metabolic or inflammatory distress (Table 3). Also consistent with the sex-dependent effects of MIA in the glutamate pathway, *Th* and glutamate receptor *Grm4* expression was lower in males exposed to gestational immune activation in the amygdala of 3-wk-old pigs (22). Likewise, rats infused with proinflammatory LPS in the ventricle had lower expression levels of *Th* in the midbrain and brainstem regions (61).

The prevalence of differentially expressed genes annotated to cilium-dependent cell motility processes (e.g., genes in the cilia- and flagella-associated protein family -Cfap- and tektin -Tekt- families) detected in the present study exemplifies the interplay of neuroendocrine and sensory processes in the hypothalamus. Hypothalamic gonadotropin-releasing hormone (GnRH) neurons participate in the neuroendocrine control of reproduction in mammals (62). The GnRH neurons undergo migration, most of which are diffusely localized in the preoptic area of the hypothalamus at birth when axonal projection processes begin. Also, cilia can act as chemo-, mechano-, and thermo-sensors (63). Consistent with the observed patterns, a brain transcriptome study of mice exposed to polyI:C during gestation detected enrichment of synaptic structure and cilia function (48). A notable finding from the present study is that the cilium-related genes had opposite profiles between gestational and juvenile immune activation across the sexes. The previous interaction indicates that the timing of inflammatory signals influences the transcriptome underlying cilium-dependent processes.

### Neuroendocrine Processes in the Hypothalamus Affected by Sex, Gestational Immune Activation, and Juvenile Distress

The detection of the enrichment of endocrine processes among the genes impacted by MIA and juvenile distress conditions is consistent with reports that gestational immune activation in female rodent offspring is associated with changes in sex steroids, early preputial separation, vaginal opening, and age at first estrus (12, 64, 65). In male rodents, LPS-elicited gestational immune activation is associated with sexual disorders, including changes in testis weight and structure and testosterone level, potentiated by the estrogen hormone estradiol (66). The premise is that gestational immune activation can alter sexual maturation by impacting the expression of genes in the hypothalamus that modulate hormonal signals (12).

The enrichment of the steroidogenesis pathway that plays a role in reproductive processes was characterized by genes overexpressed in the hypothalamus of pigs exposed to juvenile distress conditions, particularly overexpressed in fasting females and polyI:C-exposed females (Table 1). Steroidogenesis encompasses processes that produce steroid hormones, including sex steroids and corticosteroids (i.e., mineralocorticoids, glucocorticoids) and, in the present study, differential expression of genes *Fshb* and *Ptgs2*. The pattern of *Fshb* was overexpression in males exposed to juvenile distresses of inflammation or fasting (Supplemental Table S1), and high levels of FSHB are associated with low fertility in males. Responding to inflammatory insults, *Ptgs2* (an important enzyme in prostaglandin biosynthesis) was overexpressed in juvenile pigs treated with polyI:C (Table 3). Aligned with the network profile, a study of brain transcriptome in mice after peripheral bacterial challenge reported overexpression in the microglia of *Ptgs2* compared with saline-treated mice (67).

Consistent with these pathway relationships, the present study detected enrichment of response to corticosterone and mineralocorticoids characterized by the differential expression of genes in the hypothalamus responding to gestational immune activation, juvenile distress, and their interaction (Table 2). The nature of the interaction between the first and second hit observed in the present study is aligned with reports that neonatal LPS challenge is associated with decreased corticosterone response to adult stress in rats (68). Also supporting the previously observed patterns, polyI:C-elicited gestational immune activation in mice was associated with a significant increase in mineralocorticoid and, to a lesser extent, glucocorticoid receptors in the hippocampus of adult offspring (69). Mineralocorticoid and glucocorticoid receptors participate in compensatory processes triggered by chronic stress exposure known to modulate neuroendocrine responses to stress and the propensity to develop behavioral disorders. Also consistent with the present findings of the effect of juvenile distress on corticoid-related mechanisms, the stress of weaning and isolation was associated with a decrease in the expression levels of hydroxysteroid dehydrogenase and its processing products glucocorticoid and mineralocorticoid receptors in the frontal cortex of 2- to 3-wk-old pigs (70).

The observed enrichment of the prolactin pathway offers insights into the relationship between reproduction, neuronal, and inflammatory signals and their changes in response to gestational immune activation and juvenile distress. PRL participates in regulating sex drive and milk production and has inflammatory properties. PRL is modulated by the inhibitory effect of dopamine receptor 2 from the hypothalamus, whereas the secretion of PRL is enhanced by the sex hormone estrogen and neuropeptide VIP (71). The present study confirmed these molecular relationships through the previously discussed enrichment of the dopaminergic synaptic transmission pathway and the differential expression of *Vip*.

Many prolactin pathway genes, including *Prl*, *Lhb*, and *Cga,* were overexpressed in the hypothalamus of pigs from control mothers (not exposed to maternal immune activation), fasting, or males (Table 3). The pattern of these genes in the hypothalamus of 60-day-old pigs coincides with that observed in the amygdala of 3-wk-old pigs exposed to gestational immune activation (22). The relationship between the profiles may be associated with *Cga* producing the α subunit of the luteinizing hormone, whereas *Lhb* produces the β subunit.

Several prolactin pathway genes, including *Prl*, *Cga*, *Socs1,* and *Socs3,* were overexpressed in juvenile pigs exposed to polyI:C. In agreement with these patterns, *Prl* was overexpressed in the hippocampus of female mice injected with *Chlamydia muridarum* (72) and after proinflammatory insults such as brain injury (73). PRL has neuroprotective or neurotrophic effects, leading to the development and maturation of dopaminergic neurons and increased proliferation of immune cells (73). Also, a study of the hypothalamic prolactin mechanism in ovariectomized rats suggested that the upregulation of suppressor of cytokine signaling SOCS proteins may aid in terminating acute signaling events, facilitate cells remaining sensitive to further prolactin signaling, and alter the reproductive effects of cytokines with the result influenced by the balance of the signals (74).

### Inflammatory Signaling and Cell Cycle Processes in the Hypothalamus That Are Affected by Sex, Gestational Immune Activation, and Juvenile Distress

The enrichment of immune response system processes among the differentially expressed genes was anticipated because of the proinflammatory signals involved in the viral gestational and viral-mimetic juvenile challenges and anti-inflammatory signals that fasting can elicit (75). The activation of the innate immune system results in higher proinflammatory cytokine levels that can be detrimental to neuronal survival and synaptic plasticity. The enriched immune-related GO biological processes and KEGG pathways include response to type I interferon, interleukin-12 production, interleukin-17 signaling, RIG-I-like receptor signaling, tumor necrosis factor, and nuclear factor kappa B signaling (Tables 1 and 2). The shared gene profiles in the type I interferon and interleukin-12, interleukin-17, RIG-I-like, tumor necrosis factor, and nuclear factor kappa B signaling processes included overexpression in juvenile pigs exposed to polyI:C, and among this group, a synergistic interaction with exposure to gestational immune activation (Table 1). This pattern is consistent with the overexpression of genes annotated to the type-1 interferon system in the hippocampi of 6- to 10-wk-old mice in response to viral infection (76), in the interleukin-17 process observed in the ventral hippocampus of adolescent mice exposed to polyI:C during gestation (77), and in the hippocampus of pigs exposed to gestational and juvenile inflammatory signals (7).

Similarly, viral intravenous infection of adult mice resulted in the induction of RIG-I and RIG-I receptor genes in the brain (78). The previous pattern was linked to RIG-I being responsible for sensing viral infections, and in the present study, polyI:C is a synthetic analog of double-stranded RNA, a molecular pattern of viral infections. Moreover, the interleukin-17 pathway was enriched among the differentially expressed genes across two studies of the cerebellum of children with autism spectrum disorder that has been associated with prenatal immune activation (79).

The enrichment of the inflammation pathway of the tumor necrosis factor exemplifies the association between the enrichment of neural signaling and inflammatory functional groups. The tumor necrosis factor modulates the trafficking of neuronal glutamate and γ-aminobutyric acid neurotransmitter receptors, therefore participating in synaptic plasticity, another previously discussed enriched process. The profile is in agreement with reports of the nuclear factor kappa B transcriptional complex capability to regulate the transcript levels for cytokines, which are elevated in the prefrontal cortex of patients diagnosed with schizophrenia spectrum disorder and mouse and primate models of gestational immune activation (80). Gestational immune activation elicited by polyI:C model of MIA can result in elevated tumor necrosis factor in adult offspring (81).

Within the enriched immune-associated pathways, differentially expressed genes included *Ccl2*, *Cxcl8*, *Il2b*, *Ifi6*, *Bst2*, *Isg15*, *Isg20*, *Oasl*, *Rsad2*, *Ccl4*, *Cxcl10*, *Il18*, interferon regulatory factor 7 (*Irf7*), *Irf1*, and *Nfkbia.* These genes were overexpressed in the group exposed to inflammatory distress and in the group exposed to gestational immune activation followed by distress (Table 3). These profiles were expected because many genes encode proinflammatory molecules that participate in the immune response and modulate the central nervous system functionality, including synaptic plasticity (82). Among the previous genes, *Ccl2*, *Cxcl8*, *Isg20*, *Oasl*, *Rsad2*, *Ccl4*, and *Cxcl10* were overexpressed in the hippocampus of pigs exposed to gestational immune activation and postnatal distress at 2 mo of age (7). Also consistent with our findings, the prefrontal cortex of rats exposed to gestational immune activation elicited by polyI:C presented overexpression of cytokines and *Isg15* (83), a gene also overexpressed in the present study of pig hypothalamus (Table 2).

In agreement with the observed gene profiles, the expression of genes *Ccl2* and *Nfkbia* was higher in the brain of a rodent model that overexpresses human tumor necrosis factor α that cannot enter the brain but results in chronic peripheral inflammation (84). Also, a transcriptome study of neuron cultures after viral infection detected upregulation of *Ccl4*, *Cxcl10*, *Oas1*, *Irf1*, and *Irf7*, an important modulator of the type I interferon process (85).

Several functional categories associated with cell energy requirements and cell cycle were enriched among genes impacted by metabolic or inflammatory distress at 60 days of age (Table 2). Enriched GO biological processes and KEGG pathways included oxidative phosphorylation, ribosome, positive regulation of lipid metabolic process and transport, and cell apoptosis (Table 2). Apoptotic processes and lipid biosynthesis were also detected in a meta-analysis of brain transcriptome studies from adult old mice exposed to intermittent fasting relative to ad libitum feeding (86). Likewise, cerebellum transcriptome studies of rat (87) and mouse (88) models of ischemia reported that intermittent fasting inhibited apoptosis and enhanced neuroprotection, evidenced by the enrichment of apoptosis, glutamatergic, dopaminergic, oxidative phosphorylation, and ribosome pathways.

In addition to apoptosis, the detected enrichment of the oxidative phosphorylation and ribosome pathways among the genes impacted by prenatal and juvenile distress (Table 1) can be linked to the enriched process response to corticosterone. In the present study, genes in the oxidative phosphorylation pathway were overexpressed in pigs exposed to distress and gestational immune activation. Corticosterone is a mediator of stress response and neuronal treatment, with this hormone increasing mitochondrial oxidation potential and offering neuroprotection against stress (89). The enrichment of the oxidative phosphorylation and ribosome categories was also observed in the amygdala, hippocampus, and pituitary gland of 3-wk-old pigs exposed to gestational immune activation and weaning stress (4, 21, 44). The correlated expression profile of the genes in the oxidative phosphorylation and ribosome pathways observed in the present study is supported by findings that neurodevelopmental pathologies associated with gestational immune activation, such as autism spectrum disorder, present aberrant ribosomal protein gene expression in the brain, at the same time as abnormal energy-related oxidative phosphorylation, respiratory, and electron transport chain functionality (90).

### Conclusions

The present study demonstrated that immune activation during gestation and juvenile inflammatory or metabolic distress ∼100 days after the first hit impacted sensory, neuroendocrine, and immune response signaling processes in the hypothalamus, and some of these effects were sex-dependent. Notably, differentially expressed genes were predominant in categories including sensory perception of pain, dopaminergic synaptic transmission, glutamate receptor signaling, neuropeptide signaling, cilium-dependent cell motility, response to corticosterone and immobilization stress, steroidogenesis, endocrine system development, prolactin signaling, and feeding and reproductive behavior.

The current study uncovered hypothalamic gene profiles and pathways uniquely impacted by gestational and juvenile inflammatory and metabolic stressors, including sensory pain perception, steroidogenesis, and cilia-related pathways. The dysregulated genes included *Oprm1*, *Oprd1*, *Ptgs2*, *Fshb*, and genes in the Cfap and Tekt families. In addition, the dysregulation of other genes annotated to broad immune response and neuronal signaling processes, including dopamine, IL-17, and TNF signaling, and *Th*, *Ccl2*, *Isg20*, and *Oasl* paralleled changes observed in the hippocampus and also in the hypothalamus of 3-wk-old animals experiencing weaning stress.

The findings confirm the protracted effects of exposure to inflammatory signals in utero and the interplay with later-in-life challenges on the transcriptome of the hypothalamus. These results also aid in explaining the disruption caused by the factors on the pituitary and adrenal gland components of the HPA axis. The comparison of response to gestational and juvenile hits against baseline conditions further the characterization of the role of the hypothalamus on the sensory, feeding, and reproductive behaviors and immune and physiological responses. This information supports subsequent preclinical studies of the sex-dependent impact of inflammatory and metabolic challenges across an individual's age.

## DATA AVAILABILITY

The source data can be found in the National Center for Biotechnology Information GEO repository under Accession No. GSE245146.

## SUPPLEMENTAL DATA

10.13012/B2IDB-8635710_V1 Supplemental Fig. S1 and Supplemental Tables S1–S3: https://doi.org/10.13012/B2IDB-8635710_V1.

## GRANTS

This work was funded by United States Department of Agriculture (USDA) NIFA AFRI Grant 2018-67015-27413 (to S.L.R-Z., R.A.N., L.R., S.B., and R.W.J.), USDA NIFA Grant 2022-38420-38610 (to S.L.R-Z. and A.N.G.), and National Institutes of Health Grant P30DA018310 (to B.R.S. and S.L.R-Z.).

## DISCLOSURES

No conflicts of interest, financial or otherwise, are declared by the authors.

## AUTHOR CONTRIBUTIONS

S.L.R-Z., R.A.N., A.M.A., and R.W.J. conceived and designed research; A.M.A. and L.R. performed experiments; S.B., A.N.G., and B.R.S. analyzed data; S.L.R-Z. interpreted results of experiments; S.L.R-Z. and S.B. prepared figures; S.L.R-Z. drafted manuscript; S.L.R-Z., R.A.N., A.M.A., L.R., S.B., A.N.G., B.R.S., and R.W.J. edited and revised manuscript; S.L.R-Z., R.A.N., A.M.A., L.R., S.B., A.N.G., B.R.S., and R.W.J. approved final version of manuscript.
